# Reversing resistance to counter antimicrobial resistance in the World Health Organisation’s critical priority of most dangerous pathogens

**DOI:** 10.1042/BSR20180474

**Published:** 2019-04-12

**Authors:** Henrietta Venter

**Affiliations:** School of Pharmacy and Medical Sciences, University of South Australia, Adelaide, Australia

**Keywords:** antibiotic resistance, antibiotics, efflux pump inhibitor, membrane permeability, reversal of resistance, synergism

## Abstract

The speed at which bacteria develop antimicrobial resistance far outpace drug discovery and development efforts resulting in untreatable infections. The World Health Organisation recently released a list of pathogens in urgent need for the development of new antimicrobials. The organisms that are listed as the most critical priority are all Gram-negative bacteria resistant to the carbapenem class of antibiotics. Carbapenem resistance in these organisms is typified by intrinsic resistance due to the expression of antibiotic efflux pumps and the permeability barrier presented by the outer membrane, as well as by acquired resistance due to the acquisition of enzymes able to degrade β-lactam antibiotics. In this perspective article we argue the case for reversing resistance by targeting these resistance mechanisms – to increase our arsenal of available antibiotics and drastically reduce antibiotic discovery times – as the most effective way to combat antimicrobial resistance in these high priority pathogens.

## The current status of antimicrobial resistance

**Figure 1:**
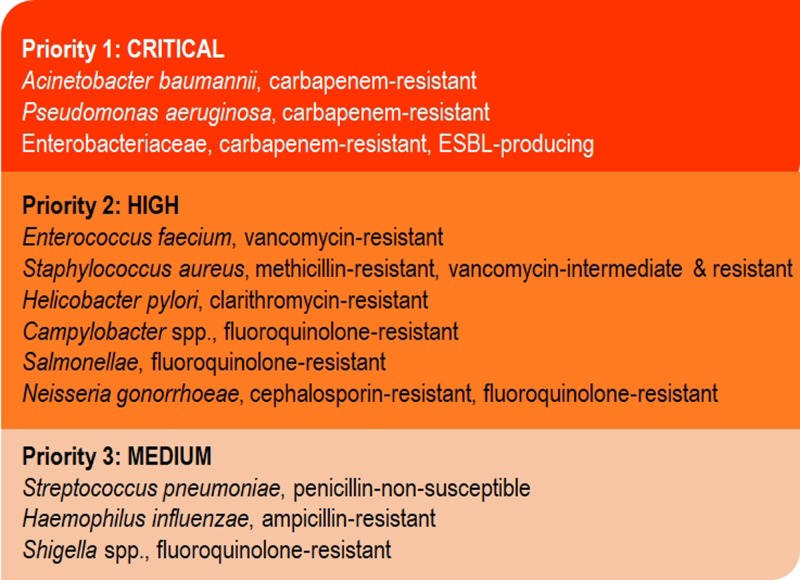
The list of the most dangerous pathogens in need of antimicrobial drug development according to the WHO [1]

**Figure 2:**
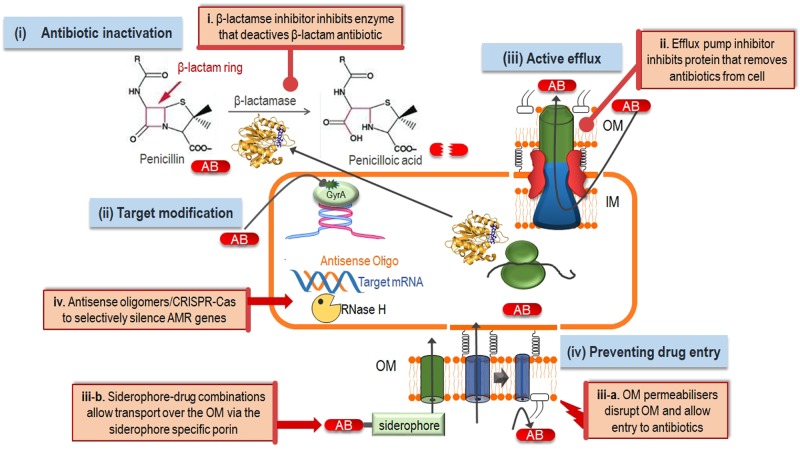
Antimicrobial resistance mechanisms in Gram-negative bacteria and ways to reverse resistance The four main mechanisms of antibiotic resistance in Gram-negative organisms (**blue boxes**) are (**i**) antibiotic inactivation, for example, the production of β-lactamase enzymes that hydrolyse the β-lactam ring thereby deactivating this class of antibiotics; (**ii**) target modification, for example modifications in the GyrA protein confers resistance to fluoroquinolones; (**iii**) active efflux, where drug efflux pumps remove the antibiotic from the bacterial cell thereby lowering antibiotic concentration to sub-toxic levels and (**iv**) prevention of drug entry through the OM by the expression of more selective porins, mutations in porins or loss of porins. Antibiotic resistance can be reversed by the addition of resistance breakers (**orange boxes**) such as (**i**) β-lactamase inhibitors to prevent antibiotic degradation; (**ii**) efflux pump inhibitors to allow the antibiotic to reach its target instead of being removed by the efflux pump; (**iii-a**) OM permeabilisers that destabilise the bacterial cell, thereby allowing antibiotics entry through the normally impenetrable OM; (**iii-b**) siderophore-drug conjugates which allow the antibiotic to breach the OM barrier by being transported through the siderophore specific porin and (**iv**) gene-silencing techniques to prevent expression of resistance determinants.

The World Health Organisation (WHO) recently published a list of antimicrobial-resistant (AMR) organisms for which the need of new therapies are the greatest (Figure 1). The most critical priority consists solely of Gram-negative organisms, specifically carbapenem-resistant *Acinetobacter baumannii*, carbapenem-resistant *Pseudomonas aeruginosa* and members of the family Enterobacteriaceae which are carbapenem-resistant and containing extended spectrum β-lactamases [1]. Infections caused by Gram-negative pathogens prove much harder to treat compared with Gram-positive organisms due to the very high intrinsic drug resistance of Gram-negatives. Intrinsic antibiotic resistance in these organisms is due to the presence of an outer membrane (OM) – which acts as a permeability barrier – and the expression of several drug efflux pumps [2–4]. Additionally, these organisms could also harbour acquired resistance mechanisms such as drug inactivation through β-lactamases that would render β-lactam antibiotics ineffective, or modification of the drug target so that the antibiotic can no longer efficiently act on that target [2] (Figure 2). Most multidrug-resistant organisms harbour several of these resistance mechanisms (e.g. [3]). However, antibiotic efflux is the predominant mechanism for aminoglycoside resistance in *P. aeruginosa* [5], fluoroquinolone resistance in *Listeria monocytogenes* [6] resistance to a variety of antibiotics in *Burkholderia* species [7] and linezolid resistance in a range of Gram-negative pathogens [8] even though the latter is available for the treatment of resistant Gram-positive bacteria. The combination of reduced OM permeability (through a lack of the OprD porin) and efflux pump expression were reported to be the main mechanisms of carbapenem resistance in *P. aeruginosa* [9].

Very limited treatment options remain for infections caused by carbapenem-resistant Gram-negatives. Hence, the ability to reverse resistance in these organisms would be of immense clinical value.

## Why is there such a shortfall in antibiotics?

Despite the well-recognised medical need for new antibiotics and the almost linear increase in antibiotic resistant infections, there has been a dramatic decrease in antibacterial drug discovery [10–12]. Many companies left the area with Novartis being the latest company to close down their anti-infective discovery pipeline (https://www.fiercebiotech.com/biotech/despite-looming-resistance-crisis-novartis-ducks-out-antibiotics-research).

The main reason for the lack of antibiotic development by pharmaceutical companies is the low return on investment [13]. Contributing factors to the lack of financial gain to be derived from antibiotic development are the short treatment times (1–7 days typically), stewardship (restricting the use of antibiotics) and short-lived efficacy before resistance start to develop (1–4 years) while the development of antibiotic is under the same stringent regulatory requirements as more lucrative drugs [14,15].

Antibiotic drug discovery pose many challenges e.g. GlaxoSmithKline used bioinformatic analysis of genomic information to identify new antibiotic targets and ran more than 70 high-throughput screening campaigns between 1995 and 2001 which did not yield a single agent to the antibiotics pipeline [16]. In fact, almost all of the antibiotics approved during the last 30 years are modifications of earlier classes of antibiotics to increase efficacy against resistant bacteria (data from the PEW charitable trusts, February 2018, https://www.pewtrusts.org/-/media/assets/2018/03/antibiotics_clinical_dev_table_february2018.pdf) [14,17] and with major drug companies exiting the field, antibiotic drug discovery now falls on academic institutions such as the Combating Antibiotic Resistant Bacteria Biopharmaceutical Accelerator (CARB-X) programme, the BEAM alliance [18], the Global Antibiotic Research & Development Partnership (GARDP) and CO-ADD community open source [19,20].

However, the trends in antibiotic development still suggest that the standard pharmaceutical and economic model will not be sufficient to address the lack of new antibiotics. While modifications of current classes of antibiotics constitutes a valuable approach, given the unique nature of these targets and consequent ability to develop agents with a high therapeutic index, we argue here the case for the development of compounds that could reverse resistance, hence reinvigorate antibiotics to which resistance has developed.

## Reversal of resistance

Compounds that reverse resistance (generally termed antibiotic adjuvants, resistance breakers, antibiotic potentiators or chemosensitisers) possess no or little antimicrobial activity themselves, but when co-administered with an antibiotic, they potentiate the activity of the antibiotic [21–23]. Most resistance breakers act by inhibiting one of the following three resistance mechanisms: (i) inhibition of the β-lactamase enzymes that inactivate β-lactam antibiotics, (ii) inhibition of antibiotic efflux pumps, (iii) acting on the bacterial OM to breach the OM permeability barrier. (iv) A completely different kind of therapy, that is not based on small molecule adjuvants, is the use of antisense-mediated gene silencing [24] or the bacterial CRISPR-Cas (clustered regularly interspaced short palindromic repeats-CRISPR-associated) immune system [25] (Figure 2).

### (i) Inhibitors of β-lactamase enzymes

β-Lactam antibiotics are among the most useful and frequently prescribed classes of antibiotics to treat bacterial infections. β-Lactams target the penicillin binding protein (PBP, a peptidyl transferase) which is a crucial enzyme needed for cell wall synthesis in bacteria. Bacteria can relatively quickly and effectively acquire resistance to β-lactams by the production of β-lactamase enzymes which cleave the β-lactam ring thereby rendering the antibiotic ineffective [2]. The use of β-lactamase inhibitors combined with a β-lactam antibiotic has been a successful strategy for overcoming β-lactamase-mediated resistance. β-Lactamase inhibitors are compounds (mostly stable β-lactams) that inhibit the β-lactamase enzymes and hence prevent antibiotic degradation (Figure 2) [26]. These inhibitors are already clinically used to great effect. For example, the combination of the β-lactam antibiotic amoxicillin and the β-lactamase inhibitor clavulanic acid is one of the most commonly prescribed antibiotics the community and hospitals [2]. A substantial amount of research and development have also been done in this field (e.g. reviewed recently in [27]. Therefore, this perspective piece would not elaborate on β-lactamase inhibitors other than an example of the newest development in the field and stating that their success is proof-of-principle that reversal of other resistance mechanisms are a viable option for treating multidrug-resistant infections.

#### New developments – Vabomere: a carbapenem and carbapemase inhibitor combination to treat complicated urinary tract infections

The carbapenem class of β-lactams are a particularly useful class of antibiotics, especially in the treatment of infections caused by multidrug-resistant Gram-negatives, as a result of their resistance to hydrolysis by numerous β-lactamases [28,29]. However, many Gram-negative organisms such as *P. aeruginosa, A. baumannii, Escherichia coli, Klebsiella pneumoniae*, etc. produce powerful carbapemases that inactivate nearly all β-lactams (including carbapenem antibiotics). The widespread dissemination of carbapemases is threatening the effectiveness of this class of antibiotics with very few treatment options remaining for serious Gram-negative infections [30]. A proven strategy to overcome resistance driven by β-lactamases is by co-administration of a β-lactamase inhibitor with the β-lactam antibiotic (Figure 2) [31–34]. Unfortunately, older inhibitors such as clavulanate are not effective against carbapemases. Vaborbactam is a novel, non-β-lactam that was specifically developed to inhibit KPC β-lactamases [35]. A combination of vaborbactam and meropenem displayed potent inhibitory activity against carbapenem-resistant Gram-negative bacteria [36,37]. In August 2017, the FDA approved the use of a meropenem–vaborbactam combination (Vabomere) for complex urinary tract infections caused by resistant Gram-negative organisms, confirming the huge potential of this method of resistance reversal when few other treatment options remain.

### (ii) Efflux pump inhibitors

Antibiotic efflux pumps are membrane proteins that actively remove antibiotics from the bacterial cell thereby lowering on-target antibiotic concentrations to sub-toxic levels (Figure 2) [38–43]. These efflux pumps are able to recognise and expel a wide spectrum of antimicrobial compounds thereby conferring multidrug resistance on pathogens including resistance against common disinfectants and last line antibiotics such as colistin [44–50]. The promiscuous substrate specificity of efflux pumps also means that other chemicals including disinfectants could select for resistance against antibiotics. For instance, an antidepressant and the chemicals found in common weed killers have been shown to select for organisms with increased resistance to clinically used antibiotics such as fluoroquinolone through the expression of antibiotic efflux pumps [51–53]. Moreover, organisms can only acquire resistance in the presence of active efflux pumps [54] and enhanced efflux of antibiotics contributes to bacterial persistence during antibiotic and other stresses [55]. Hence, efflux pumps are very attractive targets for inhibition [43,56]. Efflux pump inhibitors (EPIs) could synergise with antibiotics and resensitise bacteria to these antibiotics. This would greatly extend the arsenal of available antibiotics and also extend the lifetime of antibiotics in currently clinical use (Figure 2).

Antibiotic efflux pumps in Gram-negative organisms are large macromolecular complexes that span the inner membrane (IM), the OM and the periplasm of Gram-negative pathogens [57–59]. These drug efflux complexes are tripartite assemblies consisting of an inner-membrane protein (IMP) of the resistance nodulation cell division (RND) family, an outer-membrane protein (OMP) and a periplasmic adapter protein (PAP), which connects the first two proteins (Figure 2). The IMP catalyses drug/H^+^ antiport and is the part of the complex responsible for drug selectivity. Although Gram-negative organisms have the ability to express different classes of drug efflux proteins, the RND-type of efflux systems are the only ones that confer clinical levels of resistance [41,60–62].

The best studied example of an EPI against the tripartite antibiotic efflux pump of Gram-negative organisms is phenylarginyl-β-naphthylamide (PAβN), a simple naphthylamide peptide which did not progress beyond clinical trials due to toxicity [63]. Recent activity in this field by our group and others led to the design and synthesis of several compounds with increased efficacy [64–67] and low cytotoxicity [68].

#### The current status of EPI discovery

Several compounds that are able to synergise with antibiotics against drug-resistant Gram-negative bacteria are described in the literature. However, the rate of translation of these promising compounds into EPIs for clinical application is still low. One of the foremost reasons for poor eventual performance of promising lead compounds is due to the lack of follow-through from first identification of a compound with synergistic effects to identification of target-specific activity, and then execution of a thorough investigation into its mechanism of action. One of the most significant problems in current screening campaigns for EPIs is that in many cases the synergism observed is actually due to off-target effect such as non-specific damage to the bacterial membrane [43]. This is an important issue, as it indicates that the compound could have similar activity against mammalian cells and hence would be cytotoxic. This was clearly the case for PAβN [63].

However, our and other groups has some success with functional and structural determination of tripartite efflux pumps [57,69–71] as well as their interaction with carbapenems [72,73]. This robust understanding of assembly and efflux mechanism combined with the first inhibitor-bound structures of RND-type efflux proteins [66,74] could form a solid platform for drug discovery and development aimed at reversing resistance through efflux inhibition.

#### New developments – an EPI success story for Gram-positive infections

As is the case with all antibiotic development, the development of EPIs for Gram-negative bacteria lags behind that of Gram-positive bacteria. Many compounds that inhibit the efflux pumps of Gram-positive bacteria have been discovered and are well-progressed along the path of clinical development. Specifically, EPIs that reverse resistance in *Mycobacterium tuberculosis* have already been shown to accelerate treatment with rifampin in murine models of infection [75] which have led to the initiation of a clinical trial (Annual Report of the National Institute for Research in Tuberculosis; http://www.nirt.res.in).

### (iii) By-passing the permeability barrier

Another intrinsic mechanism of resistance in Gram-negative organism is the OM which is the first line of defence by acting as a formidable permeability barrier to prevent the entry of many antibiotics (Figure 2). The OM is an elaborate asymmetric bilayer consisting of phospholipids (inner leaflet) and lipopolysaccharides or lipo-oligosaccharides [76,77]. Large hydrophobic antibiotics can traverse the OM through passive diffusion which is a relatively slow process, while small hydrophilic compounds gain access through porins (used for uptake of nutrients) that are embedded in the OM [4,78–80]. Large hydrophobic antibiotics are excluded. Examples include compounds that are effective against Gram-positive bacteria such as vancomycin and teicoplanin.

The efficacy of antibiotics heavily relies on their ability to reach their intended targets at inhibitory concentrations. Efficient delivery of antibiotics to their bacterial targets are therefore an additional challenge in Gram-negative bacteria that should be taken into account in the development of antibiotic treatments against these infections [81]. Methods to quantify antibiotic concentrations in the periplasm or in the cytosol of bacterial cells [82–84] would therefore be a valuable tool in future antimicrobial drug discovery against Gram-negative pathogens. Additionally, the importance of porins in the uptake of antibiotic necessitate an in-depth understanding of the translocation process [78] that would facilitate the use of virtual screening techniques to search for new molecular scaffolds with enhanced permeation [85].

Several studies have reported that targeting of OM permeability can be an effective strategy for increasing antibiotic efficacy [22,86–88]. Some antibiotic screening campaigns use a ΔTolC mutant of *E. coli* as absence of the OMP TolC enhances drug sensitivity [89]. There are some merits in this approach as it would allow a higher rate of discovery of compounds with activity against various targets in Gram-negative bacteria; target delivery can then be the next step in the drug development pathway. A different approach is to deliver antibiotics in combinations with chemosensitisers that could breach the permeable barrier of the OM and so enhance antibiotic uptake.

#### OM permeabilisers

We have investigated the addition of ethylenediaminetetraacetic acid (EDTA) as chemosensitiser. EDTA is a well-known metal chelator that can cause OM permeabilisation [90] and is widely used to study e.g. bioenergetics in Gram-negative bacteria [91] and for dye-based methods to confirm IM integrity [67,68]. EDTA treatment leads to a release of LPS which is then compensated for by an increase in glycerophospholipids, resulting in patches of phospholipid bilayer with increased permeability to lipophilic compounds [76]. We have already showed that OM permeabilisation with sub-toxic concentrations of EDTA could enhance efficacy of an EPI by several fold [67] and that a Gram-positive selective new antibiotic also displayed Gram-negative activity in the presence of EDTA [92]. The safety profile of EDTA by itself is well-established as intravenous EDTA-chelation therapy is used to treat lead poisoning [93] and an EDTA chelation therapy regimen has been trailed to determine its safety and efficacy for individuals with prior heart attacks [94].

Other chemosensitisers are compounds that are used as antimicrobials such as silver, polymyxins, etc. but could be used as antibiotic adjuvants at sub-toxic levels to enhance permeation and subsequent efficacy of antibiotics [87,95]. Polymyxins are cationic cyclic lipopeptides that bind to LPS and so permeabilise the OM. These peptides re-emerged in clinics to treat multidrug-resistant Gram-negative infections. Polymyxin E, otherwise known as colistin, is now the last-resort treatment for infections caused by carbapenem-resistant pathogens [96,97]. The dose regime for polymyxins needs to be very carefully controlled due to their inherent nephrotoxicity. However, polymyxins could be used at concentrations far below their MIC to permeabilise the OM and synergise with other antimicrobials [95,98,99]. Alternatively, the non-cytotoxic polymyxin non-apeptide could be used as antibiotic adjuvant [86]. The octapeptins are another family of cyclic lipopeptides which were discovered about 40 years ago and, similar to the polymyxins, they have been largely ignored in the interim. Importantly though, octapeptin retains efficacy against polymyxin-resistant bacteria due to their interaction with both lipid A and phospholipids. Octapeptins also have a broader spectrum of activity that include Gram-positives and yeasts and displays a superior preclinical safety profile compared with the polymyxins [86,100,101]. Hence, in addition to their antibiotic activity octapeptin could also be ideal resistance breakers to be used as adjuvants to reverse resistance in the most critically important multidrug-resistant organisms. Pletzer et al. [102] reported that antibiofilm peptides also acted as resistance breakers and synergised with a range of antibiotics in an *in vivo* mouse model of infection with multidrug-resistant Gram-negatives bacteria such as *K. pneumoniae, A. baumannii, P. aeruginosa* and *Enterobacter cloacae*. At least part of this synergism was due to OM permeabilisation by the peptides.

Lipid modulation plays an important role in permeability, hence compounds that would alter the lipid composition or lipid content of the OM could be valuable chemosensitisers too. To this extent, a high-throughput analysis revealed the small molecule MAC13243 as membrane permeabiliser to facilitate increased influx of large antibiotics in *E. coli*. This molecule was identified as an inhibitor of the LolA, a periplasmic chaperone that traffics lipoproteins from the inner to the OM [103].

A ‘Trojan horse’ strategy of connecting antibiotics to iron-binding molecules (siderophores) and thereby utilising the inherent iron uptake machinery of Gram-negative bacteria to breach the OM barrier has been under investigation since the 1980s (Figure 2) [104,105]. Siderophores are high-affinity iron scavenging molecules excreted by pathogens to remove iron thereby allowing the organism to overcome iron limitation in the host. Gram-negative organisms use dedicated OM porins to allow entry to the siderophores. Importantly, the addition of antibiotics to siderophores did not seem to hamper their uptake through their respective porins. This very promising approach lead to the development of several siderophore-conjugated monobactam antibacterial agents with excellent *in vitro* activity against multidrug-resistant Gram-negative pathogens such as *P. aeruginosa* [106]. Unfortunately, further development of these particular combinations was hampered by a lack of *in vivo* activity and the quick development of resistance against the first candidate conjugates [107,108].

#### New developments – Trojan horses to deliver antibiotics over the OM

Siderophore–antibiotic combinations has been revisited, this time with clinical success. Cefiderocol (a catechol-substituted siderophore–cephalosporin combination) has excellent *in vitro* [109] and *in vivo* [110] efficacy against a range of Gram-negative multidrug-resistant organisms and is currently undergoing Phase 3 clinical trials [111].

The Gram-negative bacterial OM is not just an intrinsic resistance mechanism, but organisms can also acquire resistance through mutations in the porins through which antibiotics gain access over the OM. Hence, antibiotic adjuvants that permeabilise the OM or therapeutics designed to be transported over the OM are excellent ways to breach both these intrinsic and acquired resistance mechanisms of the OM and is gaining track as valuable treatment options to reverse resistance in multidrug-resistant Gram-negative bacteria.

### (iv) Gene silencing technologies

Gene silencing technologies for the reversal of resistance is still in its infancy with many hurdles, notably on-target delivery of these technologies, still to be overcome. The expression of resistance genes could be suppressed by either antisense oligomers [24] or by utilising the bacterial CRISPR-Cas immune system [25].

#### Antisense oligomers targeted at AMR resistance genes

Antisense oligomers are short, single-stranded oligomers that mimic the structure of DNA or RNA. Based on the chemistry of the sugar-phosphate backbone the antisense oligomers can be divided into RNase H-incompetent or RNase H-competent. RNase H-incompetent antisense oligomers bind to the target RNA and prevents binding of the 30s Ribosome, thereby preventing transcription of the mRNA while binding of RNase H-competent antisense oligomers leads to activation of RNase H and degradation of the target mRNA. The latter approach has the distinct advantage that RNase H-dependent oligonucleotides can inhibit protein expression when targeted to virtually any region of the mRNA while the RNase H-incompetent oligonucleotides are efficient only when targeted to the 5′- or AUG initiation codon region [112]. For this reason the majority of antisense drugs investigated for clinical use are function via the RNase H-dependent mechanism. Fomivirsin, the first FDA-approved antisense therapeutic that targets a microorganism (cytomegalovirus) is also based on an RNase H-dependent mechanisms [113].

The New Delhi metallo-β-lactamase (NDM-1) is a plasmid-associated metallo β-lactamase that confers resistance to carbapenem antibiotics. Sully et al. [114] developed a phosphorodiamidate morpholino antisense oligomer targeted to the *bla*_NDM-1_ gene for the NDM-1 carbapemase. The antisense oligomer was conjugated to an arginine-rich peptide, which improves penetration of the oligomers into bacteria [114]. This peptide-conjugated antisense oligomer restored bacterial susceptibility to carbapenems and protected mice in a lethal model of sepsis when co-administered with meropenem.

#### CRISPR-Cas to selectively remove AMR genes

CRISPR-Cas is a bacterial immune system that protects bacteria against invading nucleic acids. This system is widely used for genome editing and has great potential to be utilised to selectively remove AMR genes from bacterial populations. RNA-guided nucleases target and remove specific DNA sequences and hence the system could be programmed to remove genes coding for resistance determinants. The β-lactamase coding genes *bla*_SHV-18_ and *bla*_NDM-1_ has been targeted by designed RNA-guided nucleases [115]. Similarly, Bikard et al. managed to selectively target the *mecA* gene which codes for an alternative penicillin binding protein and is the main resistance determinant in methicillin-resistant *Staphylococcus aureus* (MRSA). Using a phagemid delivery system, the authors were able to drastically reduce the level of MRSA in a mixed population of bacteria [116]. The efficacy of this system was also demonstrated *in vivo* with a mouse skin colonisation model [116].

The biggest obstacle facing gene silencing technologies is high-efficiency delivery of the genetic constructs to the bacterial cells. Antisense and CRISP-Cas has yet to reach the clinic however, provided that the issues with delivery can be overcome, these techniques hold great promise for future therapies to target resistance mechanisms in bacteria.

#### New developments – antisense oligonucleotides restores sensitivity to a last line antibiotic

Colistin is a last line antibiotic used to treat carbapenem-resistant Gram-negative infections. Worryingly, the mobile colistin resistance gene (*mcr-1*) which was first identified in a pig in China [117] has now spread world-wide and colistin resistance is on the rise. Peptide-conjugated phosphorodiamidate morpholino oligomers targeted to mcr-1 mRNA were developed and could effectively resensitise *mcr-1*-positive *E. coli* strains to polymyxins. Moreover, addition of the peptide-conjugated antisense oligomers in combination with colistin significantly reduced the bacterial count and morbidity in a mouse model of septicaemia when compared with the effect of colistin alone [118].

## Discussion

Antimicrobial resistance is now a worldwide therapeutic problem with MDR Gram-negative bacteria, which are untreatable with any current antibiotic, fast becoming a reality in healthcare settings. With most big Pharma lacking the financial incentive to address this problem, it is up to research laboratories to provide solutions. One way of accelerating antimicrobial drug discovery and development is to reverse resistance to our currently used antibiotics by co-administering resistance breakers with these antibiotics. Huge success has already been reached by the use of β-lactams in combination with β-lactamase inhibitors. However, there is ample scope for increasing the use of our current arsenal of antibiotics even more.

Inhibition of drug efflux pumps would resensitise cells to antibiotics to which it have developed resistance (e.g. efflux-mediated resistance to carbapenems and fluoroquinolines). In addition, EPIs could also render antibiotic such as linezolid that is used to treat highly resistant Gram-positives but lack efficacy against Gram-negatives due to efflux, as new treatment options for MDR Gram-negative infections.

The arsenal of available antibiotics will be greatly expanded if compounds that are currently only active against Gram-positive bacteria but share a common target with Gram-negative bacteria could be delivered to their target site. Additionally, several last-line antibiotics that are used to treat resistant Gram-positives e.g. vancomycin and the vancomycin analogues telavancin, oritavancin and dalbavancin, the glycopeptide antibiotic teicoplanin or mupirocin [95] could potentially be rendered active against Gram-negatives by using chemosensitisers to breach the OM permeability barrier. Similarly teixobactin, the only new class of drug discovered in the last 33 years [119], has high efficacy against Gram-positive bacteria but lacks any Gram-negative antibacterial activity. The target for teixobactin is lipid II [120,121] an essential precursor for both Gram-positive and Gram-negative cell wall synthesis. Hence, the Gram-positive selective activity of teixobactin could most probably be attributed to inability to reach its target in Gram-negatives; this issue could potentially be solved by co-administration of an OM permeabiliser.

Three out of the eleven antibiotic treatments currently in Phase III trials are combinations of antibiotics with molecules designed to overcome resistance (data from Pew charitable trust, www.pewtrusts.org/en/research-and-analysis/data-visualizations/2014/antibiotics-currently-in-clinical-development). As drug discovery and development are not able to keep up with the development of resistance, efforts should be made to speed up this process. In our opinion cutting the discovery time by revitalising antibiotics to which resistance have developed or to which intrinsic resistance mechanisms exist is the most sensible way of reducing the antimicrobial drug discovery and development timeline; this would be imperative for addressing the treatment void for the organisms in the WHO’s most critical priority for antibacterial drug development.

## Abbreviations

AMR: antimicrobial-resistant
EDTA: ethylenediaminetetraacetic acid
EPI: efflux pump inhibitor
IM: inner membrane
IMP: inner-membrane protein
MRSA: methicillin-resistant *Staphylococcus aureus*
NDM-1: New Delhi metallo-β-lactamase
OM: outer membrane
OMP: outer-membrane protein
PAβN: phenylarginyl-β-naphthylamide
PAP: periplasmic adapter protein
RND: resistance nodulation cell division
